# Microdroplet-guided intercalation and deterministic delamination towards intelligent rolling origami

**DOI:** 10.1038/s41467-019-13011-w

**Published:** 2019-11-04

**Authors:** Borui Xu, Xinyuan Zhang, Ziao Tian, Di Han, Xingce Fan, Yimeng Chen, Zengfeng Di, Teng Qiu, Yongfeng Mei

**Affiliations:** 1 Department of Materials Science, State Key Laboratory of ASIC and Systems, Fudan University, Shanghai, China; 2 State Key Laboratory of Functional Materials for Informatics, Shanghai Institute of Microsystem and Information Technology, China Academy of Science, Shanghai, China; 3 School of Physics, Southeast University, Nanjing, China

**Keywords:** Mechanical engineering, Nanoscale materials, Synthesis and processing

## Abstract

Three-dimensional microstructures fabricated by origami, including folding, rolling and buckling, gain great interests in mechanics, optics and electronics. We propose a general strategy on on-demand and spontaneous rolling origami for artificial microstructures aiming at massive and intelligent production. Deposited nanomembranes are rolled-up in great amount triggered by the intercalation of tiny droplet, taking advantage of a creative design of van der Waals interaction with substrate. The rolling of nanomembranes delaminated by liquid permits a wide choice in materials as well as precise manipulation in rolling direction by controlling the motion of microdroplet, resulting in intelligent construction of rolling microstructures with designable geometries. Moreover, this liquid-triggered delamination phenomenon and constructed microstructures are demonstrated in the applications among vapor sensing, microresonators, micromotors, and microactuators. This investigation offers a simple, massive, low-cost, versatile and designable construction of rolling microstructures for fundamental research and practical applications.

## Abstract (web summary)

Rolling microstructures have great potential among optics and micromechanics but on-demand construction with versatile materials remains challenging. Here Xu et al. precisely construct 3D rolling microstructures by liquid-triggered delamination in predictable manner and demonstrate their applications.

## Introduction

Three-dimensional (3D) microstructures capable of controllable properties of materials are important in a wide range of engineering applications^1–5^, such as micro/nanoelectromechanical systems (MEMS/NEMS)^6–8^, robotics^9–11^, and metamaterials^12–14^. Among several fabrication methods, rolling origami is a convenient approach to construct 3D curved or bended microstructures from planar nanomembranes through rolling process^15–17^. While rolling microstructures as tubes and helices have been widely applied in optics^18–21^, electronics^22–25^ and biology^26,27^, rolling origami towards nanomembranes is far different from real one in which a piece of paper is precisely deformed in accordance with our desire. This gap is created due to the difference from the driving force for rolling, that is, external force by hand versus internal strain inside nanomembrane. Up to date, several trials have been made towards controlling the rolling behavior using different strategies as creating the anisotropic etching process^28^, building anisotropic material systems^29^, and designing asymmetric planar geometries^30,31^, which are, unfortunately, only applicable in specific situation and lacking of precise manipulation.

In this work, we develop a generic strategy focusing on a simple fabrication method of microstructures based on rolling origami and intelligent construction based on precise manipulation of rolling direction. Spontaneous rolling of deposited nanomembranes was obtained by designing a pre-layer to create van der Waals bonding between the nanomembrane and substrate. Thus, nanomembranes with internal strain could peel off and roll with the intercalation of liquid. Patterning nanomembranes with a shadow mask followed by adding one drop of liquid simply achieves massive production of rolling microstructures. Furthermore, benefiting from the manipulation of microdroplet on a substrate, precise control of rolling direction is developed to obtain all kinds of rolling microstructures via choosing the contacted point between the microdroplet and nanomembrane, whose structure could be designed and predicted with assistance of quasi-static finite element modeling. Their potential applications are explored in the area of vapor sensing, optical cavities, mechanical micromotors, and microactuators. Our strategy for parallel production and intelligent construction of rolling 3D microstructures provides a versatile way to meet practical demands and a superior method to face advanced desires.

## Results

### 3D microstructure construction with liquid intercalation

**Fig. 1:**
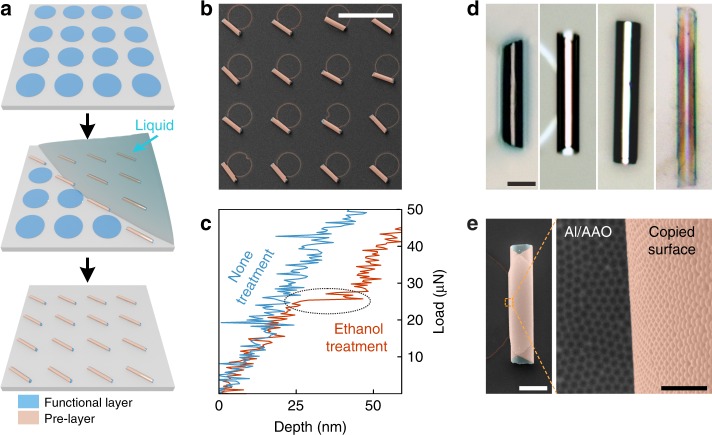
Versatile 3D rolling construction by liquid-triggered delamination. **a** Schematic illustration of parallel production of rolling microstructures by one drop of liquid. Patterned nanomembranes are rolled into 3D microstructures as liquid contacts their periphery. **b** SEM image of an array of Au/SiO/Fe microtubes on glass substrate. Scale bar, 200 μm. **c** Nanoindentation of Au nanomembrane on Si substrate with and without ethanol treatment. Adhesion is reduced with ethanol treatment. **d** Optical images of rolled-up microtubes consisting of different materials. Materials combination from left to right panels are Au/SiO/Fe/Ag on Al, Ag/SiO/Fe on glass, Ti/Cr on glass, and SiO/SiO_2_ on Au. Scale bar, 25 μm. **e** SEM images of arrayed morphology of rolled-up nanomembrane copied from Al/AAO substrate. Scale bar (left), 20 μm. Scale bar (right), 500 nm

The concept of 3D microstructure construction by liquid-triggered delamination is illustrated in Fig. 1a. Firstly, nanomembrane on the substrate was covered with a shadow mask. Shadow mask was used to define the pattern of multilayer nanomembranes. Besides, nanomembranes were designed in hierarchical structures for different purposes. The first deposited layer was applied to create van der Waals interaction with substrate, named as pre-layer. In addition, subsequent layers were deposited to generate strain gradients as well as integrate functions, named as functional layer. After peeling off the shadow mask, one drop of liquid was added on the surface. The liquid flowing on the surface triggered the rolling behavior of patterned nanomembranes as it was contacted to the periphery of nanomembranes, which led to liquid intercalation between the substrate and nanomembrane. Benefiting from the tiny amount of liquid used, 3D microstructures could maintain their hollow geometries regardless of the influence of liquid evaporation. Figure 1b is a scanning electron microscope (SEM) image elucidating successful fabrication of an array of tubular microstructures consisting of Au, SiO, and Fe, in which the Au layer was directly contacted with the glass substrate tending to delaminate with ethanol intercalation. Similarly, material exchange between the pre-layer and substrate could also lead to successful fabrication of 3D microstructures as exemplified in Supplementary Fig. 1, in which SiO/Cr bilayer microtubes were prepared on the Au substrate. Focused SEM images in Supplementary Fig. 2 further confirmed a high quality of yielded rolling microstructures. Based on this delamination phenomenon, we proposed other massive production methods of 3D microstructures in a convenient way, which are discussed in Supplementary Note 1 and Figs. 3–5.

### Mechanism of delamination and versatility of construction

In order to clarify the influence of liquid intercalation on the deposited nanomembranes, nanoindentation was used to examine the adhesion variation between the Si substrate and single-layer Au nanomembrane as an instance, of which result is present in Fig. 1c. Without any treatment, the depth is proportional to loading force, reflecting that Au nanomembrane keeps adherent on Si substrate in the testing range. On the contrary, after placing the testing sample in ethanol vapor atmosphere, scratching depth increases sharply as loading force reaches 25 μN, elucidating the delamination of Au nanomembrane from Si substrate. Hence, it is concluded that the intercalation of liquid reduces van der Waals interaction between the pre-layer and substrate, making it possible for multilayer nanomembrane to overcome the bonding and turn into 3D microstructures by means of internal strain gradient.

As aforementioned, the construction of 3D microstructures is established on the basis of spontaneous delamination of nanomembranes with van der Waals interaction to the substrate. This principle allows us to develop a generic strategy focusing on the fabrication of rolling microstructures with liquid guidance in diverse materials spanning from metals to oxides. The strategy starts with selection of material pair with van der Waals interactions to serve as substrate and pre-layer. One of the selected materials was deposited firstly to modify the surface of substrate, followed by deposition of the other material patterned with a shadow mask. Exchange between the roles of material pair could produce rolling microstructures in different material combinations, as discussed in Fig. 1b and Supplementary Fig. 1. A set of microtubes made of various material combinations is demonstrated in Fig. 1d. The first one is an Au/SiO/Fe/Ag microtube on Al substrate, while the second and third one are the microtubes on glass, which consists of Ag, SiO, Fe and Ti, Cr, respectively. Other than metal-based rolling microstructures, forth frame presents a microtube made of pure oxides as SiO and SiO_2_ on Si substrate coated with an Au layer. These series of constructions manifest not only the versatility in selecting pre-layers with corresponding substrate but also the capability in nanomembrane layer design from bilayer to multilayer, imparting the feasibility in multifunction design.

Moreover, the morphology of rolling microstructures is allowed to be further modified with fine structures with assistance of designed substrate surface. Figure 1e presents a microtube with nanodot-arrayed outer wall, which is copied from the Al substrate after the growth and etching of anodic aluminum oxide (AAO). Atomic force microscope image in Supplementary Fig. 6 validates the arrayed nanodots on the surface of curved nanomembrane. Besides, the size of the nanodots is determined by pore diameter on Al surface, which can be tuned by the anodic voltage during the growth (Supplementary Figs. 7 and 8). This nanodot-arrayed surface has great potential in tubular micromachines. On the one hand, such surface can highly accelerate the chemical reaction to generate higher boost power for tubular micromotors^32,33^. On the other hand, this tubular structure with rough surface is a perfect container to capture surrounding particles and provides excellent detection properties through surface enhanced Raman scattering^34,35^.

### Deterministic formation of tubular microstructures

Beyond the massive and versatile fabrication of rolling microstructures based on liquid-triggered rolling, precise control on microdroplet is developed to manipulate rolling direction in order to prepare desired rolling microstructures. First, nanomembranes with designed pattern were deposited, and then a capillary was used to eject single microdroplet on the surface of substrate. The motion of microdroplet on the surface was controlled by the capillary owing to the surface tension. Once the microdroplet was contacted with the periphery of deposited nanomembrane, the microdroplet triggered rolling behavior starting from the contacted point (Supplementary Fig. 9).

**Fig. 2:**
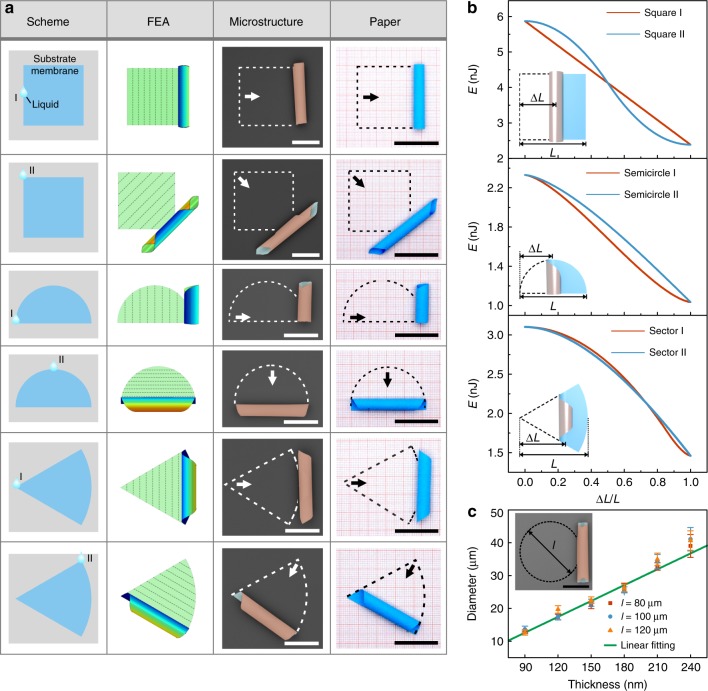
Microdroplet-guided construction of tubular microstructures. **a** The scheme of square, semicircle, and sector-patterned nanomembrane with different microdroplet trigger point, FEM predictions, experimental results (SEM images), and corresponding paper works (photographs) of six different tubular microstructures. Dashed lines in the FEM model elucidate the sketch of partition in the quasi-static model. Scale bar (third column), 50 μm. Scale bar (forth column), 3 cm. **b** Calculation results in total elastic energy change during different rolling routes of square, semicircle, and sector pattern design. The insets illustrate the geometric parameters released length Δ*L* and total released length *L*. Elastic energy is relaxed slightly differently because of different released behaviors. **c** The diameter of tubular microstructures related to the pattern size and total thickness. The inset is the SEM image of rolled-up microtubes with circle pattern, of which diameter is defined as *l*. Scale bar, 50 μm

Three groups of demonstrations of different patterns including square, semicircle, and sectors are shown in Fig. 2a. With assistance of microdroplet manipulation, various geometries of tubular microstructures were successfully fabricated by tuning the trigger point. With the aid of finite element analysis^36^ as discussed in Supplementary Note 2 and Supplementary Fig. 10, the final structures of rolled-up nanomembrane could be precisely predicted with assigned patterns and rolling direction. The second and third column of Fig. 2a present the finite element method (FEM) results and SEM images of final structures, respectively. Besides, the procedure of triggered rolling was recorded in Supplementary Movies 1–3. In the first demonstration, a square-patterned nanomembrane was rolled into microtube with smooth or tipped ends by moving the microdroplet to the side or vertex of the square. In another example, a semicircle-patterned nanomembrane was constructed into microtube with asymmetric or symmetric ends by triggering rolling behavior from the corner of the semicircle or the midpoint of the arc. The same situation was also obtained on sector-patterned nanomembrane. Similarly, we prepared origami work of paper according to the corresponding pattern and rolling direction, reflecting that deterministic delamination by microdroplet guidance is an effective and efficient tool in the 3D microstructure construction just as we did to a piece of paper with our hands.

Figure 2b gives the calculated results of the elastic energy (*E*) related to the relative released distance (released distance versus total length, ∆*L*/*L*, illustrated in the insets of Fig. 2b) to clarify the energy difference in rolling process along different directions. According to the fact that both rolled-up microtubes with the same pattern design in Fig. 2a are made of same material system and equal in final diameter, released bending energy density, and final elastic energy density of both directions are the same, respectively (details can be found in Supplementary Note 3). The elastic energy variation is only influenced by the area change of released nanomembrane, of which energy density changed from planar to rolling. So it is obvious that elastic energy is relaxed slightly differently because of different released behaviors, which leads to different paths of area release. In addition, total energy of the material system was analyzed in Supplementary Note 4 and Supplementary Fig. 11, which reflects that liquid treatment overcomes the energy barrier of delamination and strain relaxation, leading to an ultrafast energy release similar with a snap-through behavior^37,38^.

**Fig. 3:**
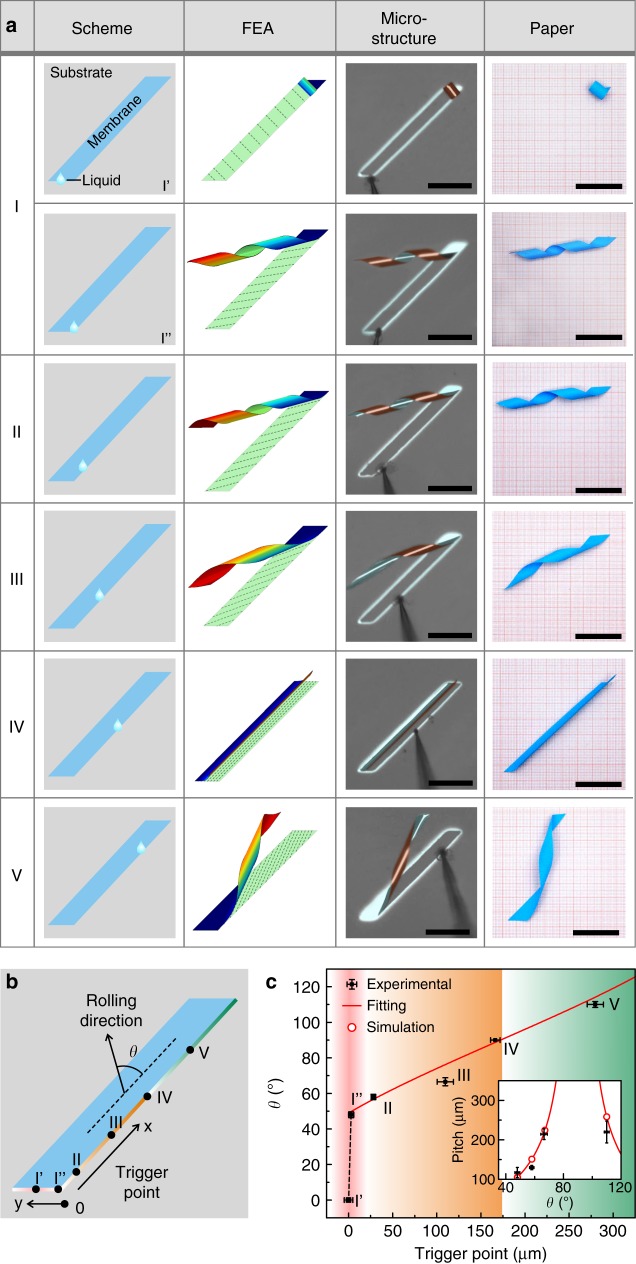
Precise control in rolling direction towards helical microstructure construction. **a** The scheme of parallelogram-patterned nanomembrane with different microdroplet trigger point (I–V), FEM predictions, experimental results (optical images), and corresponding paper works (photographs) of six different microstructures. Dashed lines in the FEM model elucidate the sketch of partition in the quasi-static model. Scale bar (third column), 100 μm. Scale bar (forth column), 3 cm. **b** Schematic illustration of the definition of rolling angle *θ* and trigger point in parallelogram-patterned nanomembrane. Colorized edges depict different rolling behavior starting from the right bottom corner (orange) and right top corner (green). **c** Relation between rolling angle *θ* and trigger point. The diagram is divided into three regions according to different rolling behavior in **a**. The inset plots the relation between helical pitch and rolling direction. Error bars represent standard deviation

As the rolling of delaminated nanomembranes is driven by internal strain, the size of obtained microstructures is determined by elastic properties and strain gradient of deposited materials rather than the intercalation of liquid. The tunability of the size is investigated in Fig. 2c. A circle pattern was applied to exclude the asymmetric geometry effect on rolling behavior^30^. Au, SiO, and Fe were deposited in the same thickness. For instance, 90 nm refers to 30 nm for each layer, and 240 nm refers to 80 nm. Besides, the size of circle pattern was set as an additional parameter defined as *l*, which is illustrated in the inset of Fig. 2c. Statistical diameters were plotted in Fig. 2c based on ten rolled-up microtubes for each parameters. The result reflects that diameter rises with increasing thickness, while the pattern size has negligible influence. In addition, according to the elastic mechanism^39^, as the thickness of each layer is equal, the diameter of rolled-up microtubes is proportional to the total thickness (details can be found in Supplementary Note 5). Thus, a linear fitting was applied as the green line in Fig. 3b and it matches well with experimental results. Therefore, it is proved that the size of microstructures can be tuned by deposition parameters and designed with theoretical calculation.

### Deterministic formation of helical microstructures

The precise control in rolling direction permits intelligent construction of more complex rolling microstructures as helix. In previous reports, it is common to construct anisotropic materials systems^40,41^ or strain^42,43^ to obtain helical microstructures. Besides, helical microstructures based on isotropic materials were achieved through asymmetric pattern design^44^. This deterministic delamination method for rolling microstructures provides a much simple tool to construct helical microstructures of isotropic materials with isotropic strain gradient, also getting rid of limited materials and complex fabrication procedures.

To illustrate the capability in the fabrication of complex microstructures, a set of trigger points along the edge of a parallelogram-shaped nanomembrane was designed as present in the first column of Fig. 3a. The interior angle is set as 45°. The reason for picking a parallelogram shape design is that this geometry provides a better construction of various helical structures through rolling, though similar situation could be obtained in rectangular nanomembrane (Supplementary Fig. 12). Moreover, resultant 3D microstructures with FEM results are summarized in the second and third column, and corresponding experimental processes are provided in Supplementary Movie 4. The first frame shows a rolled-up microtube with several turns as the nanomembrane was curved starting from the bottom left corner, and rolled along the long side. Then, with a slight change of trigger point, nanomembranes were rolled towards the direction nearly vertical to the short side, resulting in a helical microstructure instead of microtube, as illustrated in the second frame of Fig. 3a. The third and fourth frames present two examples in which the nanomembranes were rolled into microhelices with different pitches due to different rolling directions. Besides, curved nanomembranes rather than microtubes were formed as the nanomembrane was forced to roll along the short side in the fifth frame. The last one provides another situation in which the upper part was released first to construct a microhelix in a different manner. All structures modeled through FEM with proper partition are in agreement with experimental results. Furthermore, benefiting from the quasi-static FEM that could simulate the rolling of nanomembrane step by step, the process of rolling could be perfectly modeled as we relate every step to time (Supplementary Fig. 13). Demonstrations of corresponding structures made by paper rolling are present in the fourth column of Fig. 3a.

As the trigger point was moved along the right side, different rolling microstructures were constructed ranging from microtubes and curved nanomembranes to microhelices. To better understand the way how trigger point controls rolling direction, we defined two parameters to illustrate this situation as shown in Fig. 3b. One is the angle *θ* as the angle between rolling direction and long edge of parallelogram pattern, which would highly determine the geometry of rolling microstructure. The other one is the distance from the bottom corner to trigger point to quantify the position of trigger point and its maximum value is $200\sqrt 2 \,{\mathrm{\mu m}}$ (≈282.4 μm) in this situation. Besides, the long side was divided into two regions in different color referring to different rolling behaviors, that is, rolling from bottom corner and from top corner. The relation between *θ* and trigger point was plotted in Fig. 3c according to the results in Fig. 3a. As the trigger point is around zero (red area in Fig. 3c), the rolling direction is tuned along *y*-axis, varying from 0° to 45° in a small range. To achieve precise control among 0° to 45°, parallelogram patterns with different interior angle were addressed as shown in Supplementary Fig. 14. It is clear that the angle *θ* increases with trigger point moving away from the bottom corner. Surprisingly, we found that there exists a simple linearity between cos*θ* and trigger point as the fitting curve in Fig. 3c, which gives an easy way to define the rolling direction through trigger point. Besides, the relation between helical pitch and rolling direction is given in the inset of Fig. 3c, which shows good agreement between experimental and analytical results. Furthermore, the size of helical microstructures is tuned simply by altering deposition parameters (Supplementary Fig. 15).

It is concluded that, in this fabrication method, a complete design of 3D microstructure includes three independent aspects as the pattern design for predefinition, the deposition parameters to determine the diameter of microstructures, and the microdroplet guidance to define the final rolling microstructures. In addition, quasi-static FEM provides a reliable prediction of the resultant microstructures based on the designed parameters.

### Deterministic formation of complex microstructures

**Fig. 4:**
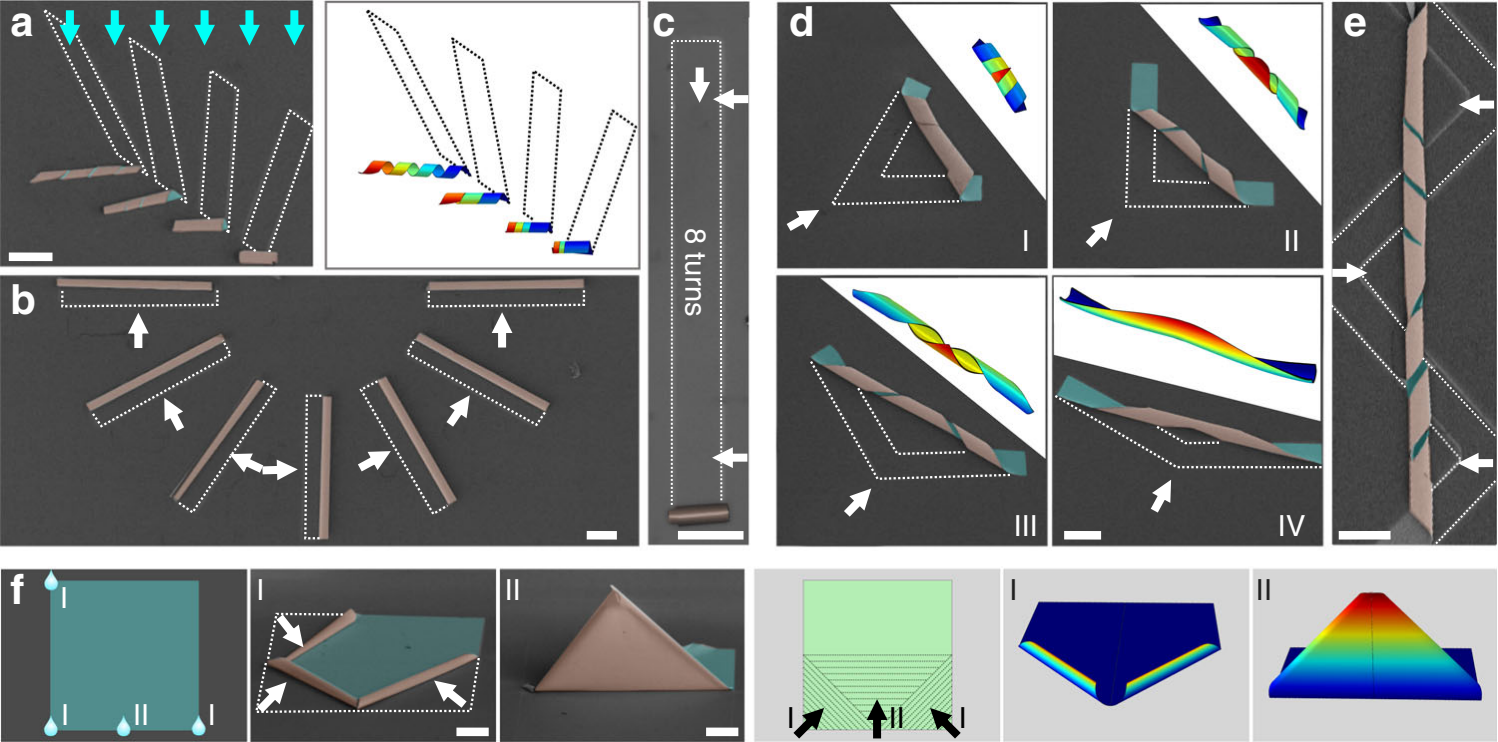
A set of microstructures assembled by deterministic delamination strategy. **a** Experimental result (left, SEM image) and FEM model (right) of different helical microstructures constructed from parallelogram-shaped nanomembranes with different interior angles. Blue arrows depict direction of liquid flow. Scale bar, 100 μm. **b** SEM image of microtubes arranged in a circle. White arrows depict rolling direction of nanomembranes. Scale bar, 100 μm. **c** Optical image of rolled-up microtubes with 8 turns. Scale bar, 100 μm. **d** Experimental results (SEM image) and FEM model of helical frameworks with opposite chirality, pitch of which was tuned by the angle between two ribbons. Scale bar, 100 μm. **e** SEM image of ultralong helical framework with different chirality constructed from zig-zag-shaped nanomembrane. Scale bar, 100 μm. **f** Experimental results (left, SEM images) and FEM model (right) of folding microstructure constructed by multi-step deterministic delamination (I and II). Scale bar, 100 μm

As aforementioned, the final 3D microstructure is able to be carefully designed with several aspects. Unfortunately, it is noticed that this design strategy is time-consuming as only one microstructure is fabricated with microdroplet manipulation. Therefore, except from control of trigger point, different microstructures were constructed through various pattern designs as shown in Fig. 4a. A series of parallelogram patterns were designed with interior angle as 30°, 45°, 60°, and 75°. The rolling direction of these nanomembranes was defined uniformly by liquid flowing along blue arrows, resulting in the formation of several helical microstructures with different pitches. This result enlightens us to achieve massive production of different rolling microstructure through pattern design and rotation, along with manipulation of liquid flow.

To emphasize the capability of this fabrication method, a set of complex microstructures was proposed. In Fig. 4b, rectangular-shaped nanomembranes were rolled along the short side to form long microtubes with a circular arrangement. In addition, this fabrication method allows successful delamination of ultralong nanomembranes, leading to a rolled-up microtube in several turns as present in Fig. 4c. It is a simple way to construct a hierarchical tubular microstructure with potential in electronic microdevices^45^. To mimic the shape of natural plants with filaments^46^, a V-shaped pattern was designed as the trigger point was set at the corner in Fig. 4d. Nanomembranes were delaminated and rolled into helical frameworks with two opposite chirality helices, in which the geometries were determined by the degree between two ribbons. Similarly, Fig. 4e illustrates a 3D zig-zag helical framework constructed from a 2D zig-zag nanomembrane. The delamination procedure was triggered at the corners of zig-zag route one by one.

Furthermore, beyond rolling into tubular or helical microstructures, a prototype of folding microstructure was developed through multi-step delamination of nanomembrane. Generally speaking, folding process requires two rigid parts without deformation and a soft part between them for bending^47,48^. As the spontaneous delamination and rolling are established on the strain gradient in deposited nanomembrane, it seems impossible to create undeformed rigid part. We circumvent this challenge with an improved control of delamination process as elucidated in Fig. 4f. Here, three microdroplets controlled by three independent capillaries were applied to simultaneously trigger the delamination of square nanomembrane from three corners, resulting in three rolled-up microtubes. Delaminated parts kept rolling until the microtubes met each other. Considering the fact that the rigidity of the microtubes is much higher than that of the undeformed part, the polygonal shape is stiffened by the formation of microtubes. Therefore, weak points with low rigidity were created at the intersection of microtubes. These parts serve as the hinges where bending happens. The next step to finish the folding behavior is using one droplet in larger volume to trigger the delamination of whole nanomembrane from literal side. And this folding microstructure could be maintained under the internal strain relaxation. This folding process is also excellently simulated by a two-step quasi-static FEM as right frames in Fig. 4f. This demonstration opens up a tantalizing prospect to release complex folding microstructure with controlled delamination of vertical asymmetric hierarchical nanomembrane, and even more advanced folding microstructures with bistability^49^.

### Demonstration of applications

**Fig. 5:**
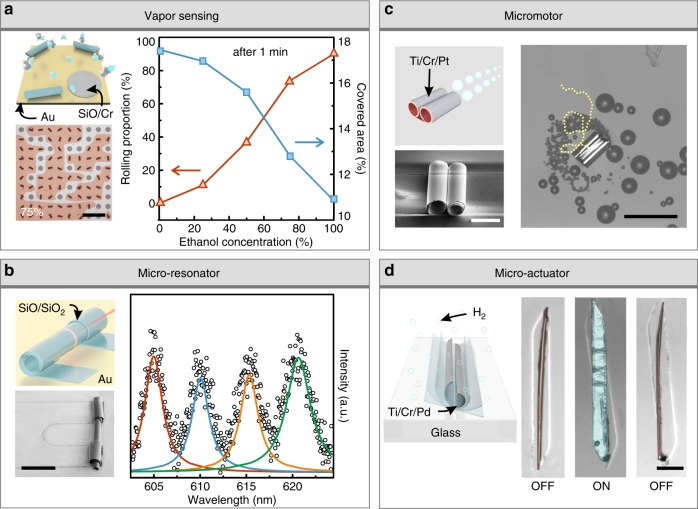
Extended construction and applications of deterministic rolling microstructures. **a** Vapor sensing based on spontaneous rolling of patterned nanomembranes. Optical image is an array of 100 nanomembranes under vapor of ethanol–water mixture in 75% concentration for 1 min. Diagram depicts the vapor sensing properties of ethanol concentration recognized by rolling proportion and covered area change. Scale bar, 500 μm. **b** Rolled-up microcavities constructed by deterministic rolling of U-shape patterned nanomembrane. Optical image is free-standing microcavity consisting of SiO and SiO_2_. Diagram is the PL spectra of the microcavity. Scale bar, 100 μm. **c** Micromotors with two tubes constructed by simultaneously triggered rolling. SEM image is the micromotors consisting of Ti, Cr, and Pt. Optical image depicts the motion of micromotor in 5% hydrogen peroxide solution, in which the yellow line is the trajectory of motion. Scale bar (SEM), 20 μm. Scale bar (optical), 100 μm. **d** Microactuators triggered by hydrogen absorption. Optical images depict the actuation behavior before (left), with, (middle), and without (right) hydrogen stimuli. Scale bar, 100 μm

The versatility and flexibility in materials and geometries of microdroplet-guided delamination approach with computational prediction suggests its infinite potential in 3D microsystems. Here, we present three demonstrations in different areas benefiting from different aspects of this approach. In Fig. 5a, a non-electrical visual vapor sensor was established based on the liquid-triggered delamination. Except from the droplet on the surface, vapor atoms in surrounding milieu also have the ability to reduce the interaction between substrate and pre-layer, which is highly influenced by the component of vapor. In another word, the component of vapor can be recognized by the rolling situation of patterned nanomembranes as illustrated in the scheme of Fig. 5a. In this demonstration, an array of SiO/Cr bilayers on Au substrate serves as a sensor to recognize the ethanol concentration of ethanol and water mixture. Optical image in Fig. 5a and Supplementary Fig. 16 present 100 nanomembranes after placed in different mixture atmospheres for 1 min. It is noticed that the proportion of rolled-up nanomembranes relates to the ethanol concentration in surrounding atmosphere. Collected data were plotted in Fig. 5a, reflecting that the rolling proportion rises from 0% with increasing concentration, which reaches 90% with ethanol vapor. Consequently, the area of surface covered by nanomembrane decreases with more ethanol, resulting in visually recognizable change of the sample. Furthermore, we explored this sensor to test the humidity as shown in Supplementary Fig. 17, which reveals that deposited nanomembranes only roll as the humidity rises to a high level. This irreversible vapor sensing can serve as an environment guarantee in the transportation and storage of expensive products.

The second demonstration is based on the rolling control of pattern-designed nanomembrane for profound optical microcavity. As present in the scheme of Fig. 5b, a U-shaped pattern was used to construct rolled-up microtubes with free-standing middle part, which was supported by other part rolling in more turns. Successful fabrication of SiO/SiO_2_ bilayer microtube on Au substrate was shown in the SEM image, as a result of the directional rolling from left to right. Photoluminescence (PL) spectrum elucidates the whispering gallery modes in the microresonator, of which the *Q* factor over 250 was obtained^19^. Although this is a very primary presentation in optics, further investigation and improvement of the deterministic delamination method for rolling microstructures would provide more complex structures with fruitful optical properties in advanced optical applications^50–52^.

In Fig. 5c, we proposed the improved fabrication method in Fig. 4f to construct complicated microstructures for micromotors. Rather than single microdroplet to determine the rolling behavior along one direction, two microdroplets controlled by two independent capillaries were applied to force the deposited nanomembrane to simultaneously roll along two directions, resulting in 3D microstructures containing two microtubes. As platinum was deposited on the top of nanomembrane as functional layer, both tubes could eject bubbles to provide thrust power, resulting in a higher speed^53^. In our experiment, the speed of micromotors with two tubes was 39.8 μm s^−1^, while the one of single tube micromotor was 20.0 μm s^−1^. Additionally, moving direction of the micromotors with two tubes can be tuned by manipulating the reaction speed in one of both, which is easily realized by localized heat or light^54^. The difference of reaction speed in our micromotor was obtained through asymmetric geometry of the tubes, leading to a spiral moving trajectory rather than linear path of a tubular micromotor as shown in Supplementary Fig. 18.

Moreover, Fig. 5d presents a microactuator through the combination of rolling microstructure and smart materials. Deposited nanomembrane was made of Ti, Cr, and Pd layers, in which Pd generates external strain through volume expansion in hydrogen environment. A tubular microstructure was constructed as the parallelogram-shaped nanomembrane was released from the substrate. It is observed that the 3D microstructure turned into 2D planar status as 4% hydrogen mixture was injected, and returned back after hydrogen desorption. This actuation behavior is dramatic and reversible, which would suggest a strategy to integrate high-density 3D mesoscale architectures into functional devices and systems.

## Discussion

In conclusion, intelligent construction of rolling microstructures was realized via the deterministic delamination triggered by liquid intercalation. This method achieves parallel production of rolling microstructures with assistance of a tiny amount of liquid, permitting a wide choice in material combinations by different substrates with pretreatment. More importantly, rolling direction of nanomembranes with isotropic properties is precisely controlled via microdroplet manipulated by capillary. Combined with pattern design and deposition control, a series of microtubes and microhelices in different geometries and sizes were constructed. Furthermore, we proposed a complete design process of 3D microstructure that is divided into three independent steps. The strain engineering including material combination and deposition parameters determines the size of structure. 2D pattern design and delamination assignment focus on the resultant geometry of 3D microstructure. A guidance for structure design was given by quasi-static FEM simulation, which provides a visualized model of resultant microstructures.

To clarify the potential of this technique, applications among several research areas were proposed. The array of deposited nanomembranes were applied as vapor sensor benefiting from the rolling behavior triggered by vapor atoms. With 2D pattern designed into a U-shape, fabricated rolling microstructure is suitable to serve as a microresonator. Besides, we developed a simultaneous triggering method to construct a micromotor with double tube. And 3D microstructure combined with smart materials was proposed as a microactuator. The examples demonstrated here might foreshadow fruitful future of designable construction with deterministic delamination in both applied demands and fundamental investigation. Moreover, this intelligent construction of 3D microstructures is expected to be available in the fabrication of rolled-up 2D materials along the desired orientation to offer outstanding properties and applications^55,56^.

## Methods

### Massive fabrication of rolling microstructures

The Si substrate was covered by a shadow mask with circular pores. Then the substrate was deposited with Au, SiO, and Fe multilayer by electron beam evaporation of which thickness was tuned as 30 nm for each layer. After the deposition of these three layers, one drop of ethanol was added on the surface. The oxide-based microtubes consisting of SiO/Cr (80/80 nm) on the Si substrate coated with an Au layer.

To realize the versatility in materials, different substrates were chosen or the substrate was deposited with a thin layer. The selected substrate and parameters in Fig. 1d are (i) Au/SiO/Fe/Ag (30/30/30/5 nm) on Al flake, (ii) Ag/SiO/Fe (30/30/30 nm) on glass, (iii) Ti/Cr (50/50 nm) on glass, and (iv) SiO/SiO_2_ (30/120 nm) on the Si substrate coated with Au layer. The substrate with arrayed nanopores was prepared through anodic oxidation of Al. The size of the pores of AAO was tuned by anodic voltage ranging from 20 to 60 V. As the anodization was completed, grown AAO was etched by NaOH solution to leave Al substrate with rough surface. Then Au, SiO, and Fe were deposited on the surface covered by a shadow mask. The thickness was tuned as 30 nm for each layer.

### Microdroplet-guided delamination for structure construction

The substrate as glass and Si was covered by a shadow mask with designed pores (square, semicircle, sector, and parallelogram). Then the substrate was deposited with Au, SiO, and Fe multilayer by electron beam evaporation of which thickness was tuned as 30 nm for each layer. After deposition, the tip of capillary containing ethanol was placed on the surface. Due to the capillary force and surface tension, a tiny amount of ethanol was ejected between the substrate and capillary, and did not spread on the surface. Motion control of microdroplet on the surface of substrate was realized by controlling capillary through a micromanipulator. Rolling process and final structures were recorded by a CCD camera connected with an optical microscope.

### Nanoindentation

One hundred nanometer Au was deposited on the Si substrate for nanoindentation. The nanomembrane was compressed by a diamond tip with a radius of 150 nm. The compression was tuned at a constant loading rate of 2.5 μN s^−1^. In addition, the max compression was 50 μN. The load–displacement data were collected to analyze adhesion properties. After first nanoindentation test in dried atmosphere, the sample was placed in ethanol vapor environment for 30 min, and then the nanoindentation was applied again with same parameters.

### Demonstrated applications of rolling microstructures

In the demonstration of vapor sensing, the Si substrate was firstly coated with thin Au layer, followed by the deposition of SiO (80 nm) and Cr (80 nm) with a circle-patterned shadow mask to construct upper nanomembranes. To test sensing properties, the sample was placed at the interior of the lid and the lid was covered on the vessel containing ethanol mixture in specific concentration. One minute later, the sample was observed through an optical microscope to count rolling proportion and calculate the covered area by nanomembrane. As for humidity sensing, different salt solutions were used to create different humidity atmosphere and the sample was placed in it for 1 h. To construct a microcavity, a shadow mask with U-shaped holes was used to define the pattern. The Si substrate was firstly coated with Au layer, followed by deposition of SiO (30 nm) and SiO_2_ (120 nm) with shadow mask. Photoluminescence spectrum was carried out on a micro-Raman spectrometer. As for the application of a micromotor, Ti (60 nm), Cr (60 nm), and Pt (2 nm) were deposited on glass substrate covered with shadow mask designed in rectangular pores. Then, two capillaries collected with two dependent micromanipulators were precisely controlled to trigger the rolling behavior from two short sides of nanomembrane simultaneously. The rolled-up microstructures were scratched into 5% hydrogen peroxide solution and its motion was recorded by a CCD camera connected with an optical microscope. In comparison, a single microtube was prepared by rolling the nanomembrane along one direction. In the application of a microactuator, nanomembrane consisting of Pd (40 nm), Cr (5 nm), and Ti (5 nm) were deposited on the glass substrate with shadow mask. After delamination of nanomembrane, 4% hydrogen mixture (H_2_/N_2_) was applied to trigger the actuation behavior. The deformation was recorded by an optical microscope.

## Supplementary information

Supplementary Information

Description of Additional Supplementary Files

Supplementary Movie 1

Supplementary Movie 2

Supplementary Movie 3

Supplementary Movie 4

Source Data

## Data Availability

The source date underlying Figs. 2c, 3c, and 5a and Supplementary Figs. 8, 14, 15, and 17 are provided as a Source Data file. The simulation and experiment data that support the findings of this study are available from the corresponding author upon reasonable request.
