# Supplementary material for: Microdroplet-guided intercalation and deterministic delamination towards intelligent rolling origami
Source: Nat Commun. 2019 Nov 4;10:5019. doi: 10.1038/s41467-019-13011-w (PMC6828951; doi:10.1038/s41467-019-13011-w)
Supplement: Supplementary file 1 — Supplementary Information [file 41467_2019_13011_MOESM1_ESM.pdf]

Supplementary Information

Microdroplet-Guided Intercalation and Deterministic Delamination towards Rolling Origami

Borui Xu[1], Xinyuan Zhang[1], Ziao Tian[2], Di Han[3], Xingce Fan[3], Yimeng Chen[1], Zengfeng Di[2], Teng Qiu[3] & Yongfeng Mei[1]*

[1] Department of Materials Science, State Key Laboratory of ASIC and Systems, Fudan University, Shanghai, China.

[2] State Key Laboratory of Functional Materials for Informatics, Shanghai Institute of Microsystem and Information Technology, China Academy of Science, Shanghai, China.

[3] School of Physics, Southeast University, Nanjing, China.

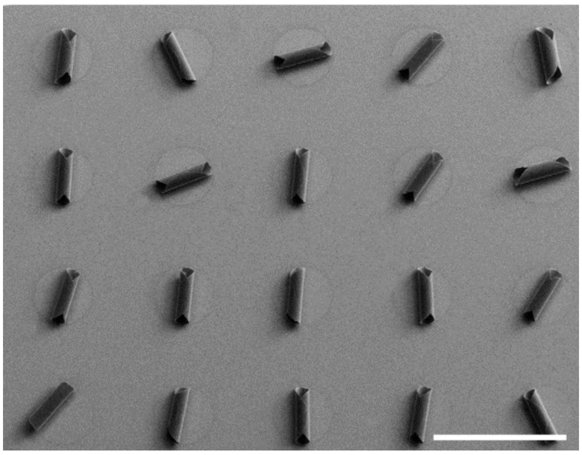

Supplementary Figure 1 | SEM image of array of oxide-based microtubes consisting of SiO and Cr on Au substrate. Scale bar, 200 μm.

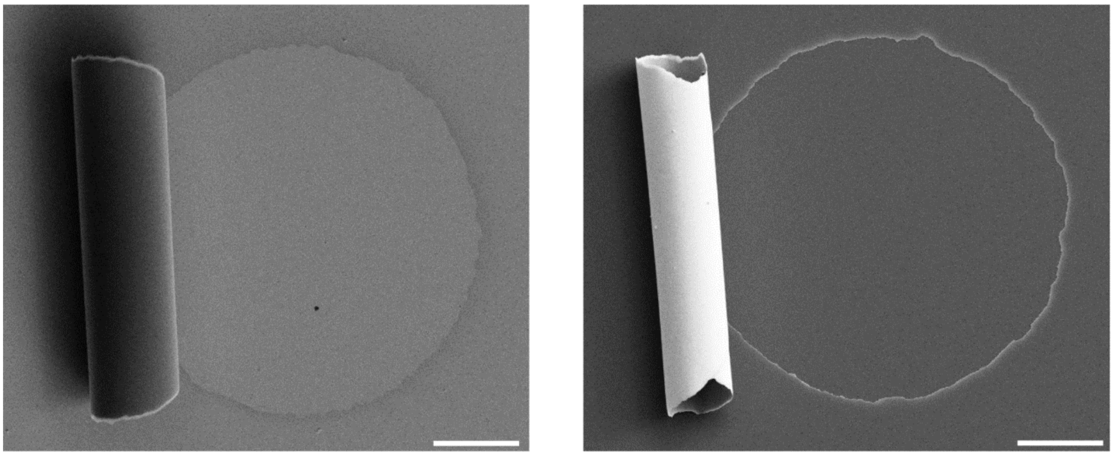

Supplementary Figure 2 | Focused SEM images of single microtube. Left one is SiO/Cr microtube on Au substrate, and right one is Au/SiO/Fe microtube on glass substrate. Scale bar, 20 μm.

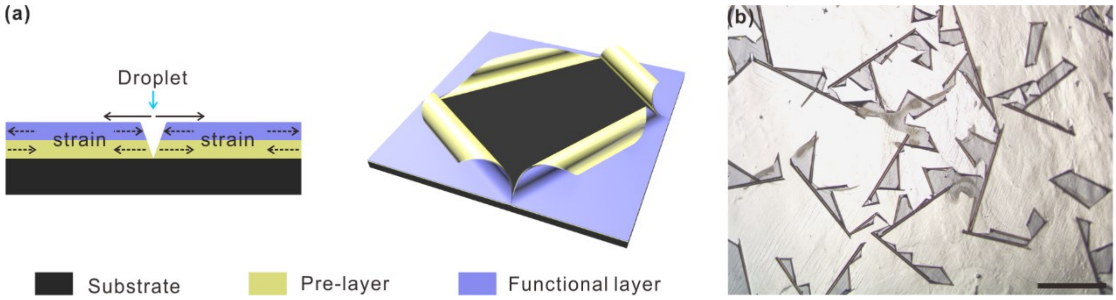

Supplementary Figure 3 | Cracking and spontaneous rolling of deposited nanomembranes with the intercalation of liquid. (a) Schematic illustration of cracking and rolling of nanomembrane owing to the intercalation of liquid. (b) Optical image of Au/SiO/Fe nanomembrane on Al substrate after the addition of one drop of ethanol. Scale bar, 200 μm.

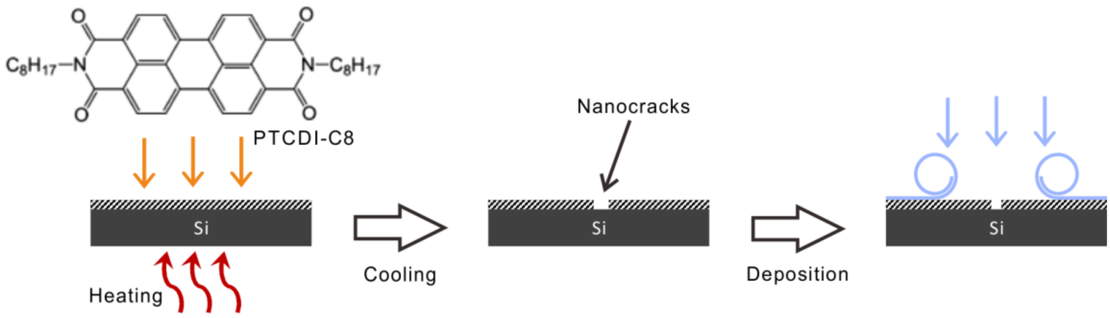

Supplementary Figure 4 | Schematic illustration of spontaneous rolling of deposited nanomembranes on nanocracked polymer substrate.

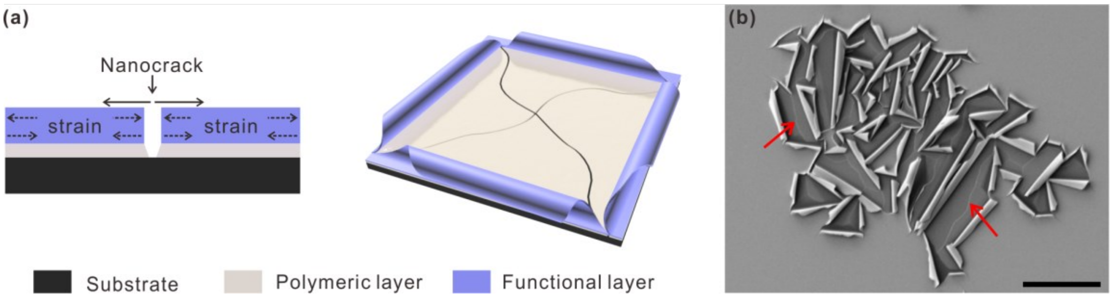

Supplementary Figure 5 | Supplementary Figure 4 | Random and spontaneous rolling of deposited nanomembrane on polymer substrate with nanocracks. (**a**) Schematic illustration of spontaneous rolling of nanomembrane starting from nanocracks. (**b**) SEM image of 30 nm Cr nanomembrane on polymer substrate with nanocracks. The red arrows point out the nanocracks on polymer. Scale bar, 20 µm.

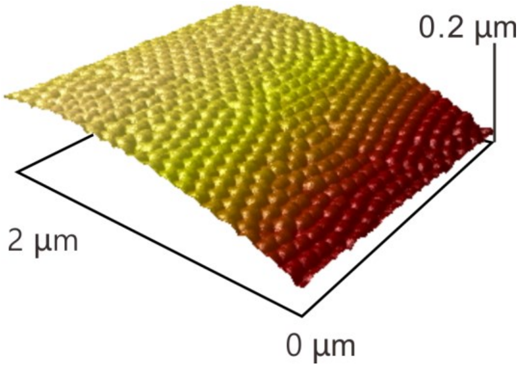

Supplementary Figure 6 | AFM mapping of arrayed morphology of rolled-up nanomembrane modified by Al/AAO substrate.

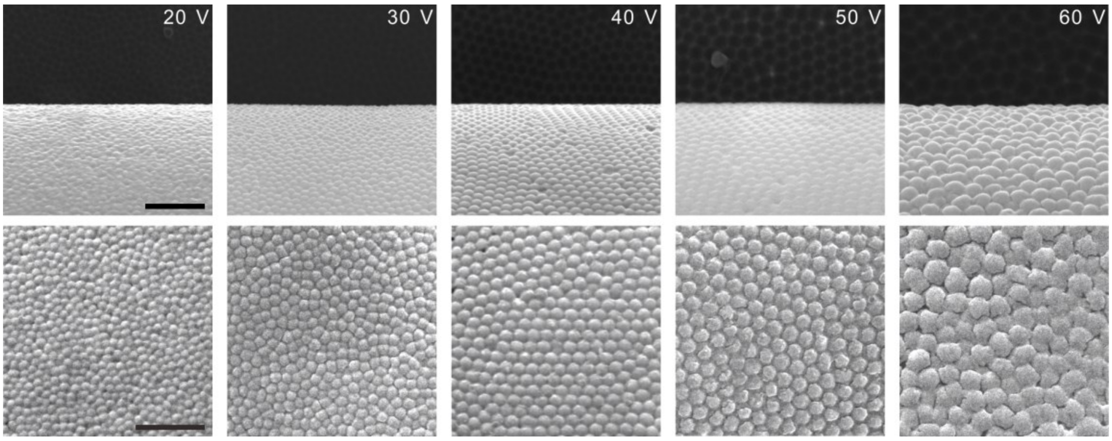

Supplementary Figure 7 | SEM images of tunable arrayed morphology of rolled-up nanomembranes. Different sizes of nanoparticles are copied from the Al substrates after anodic oxidation with various voltages. Scale bar, 500 nm.

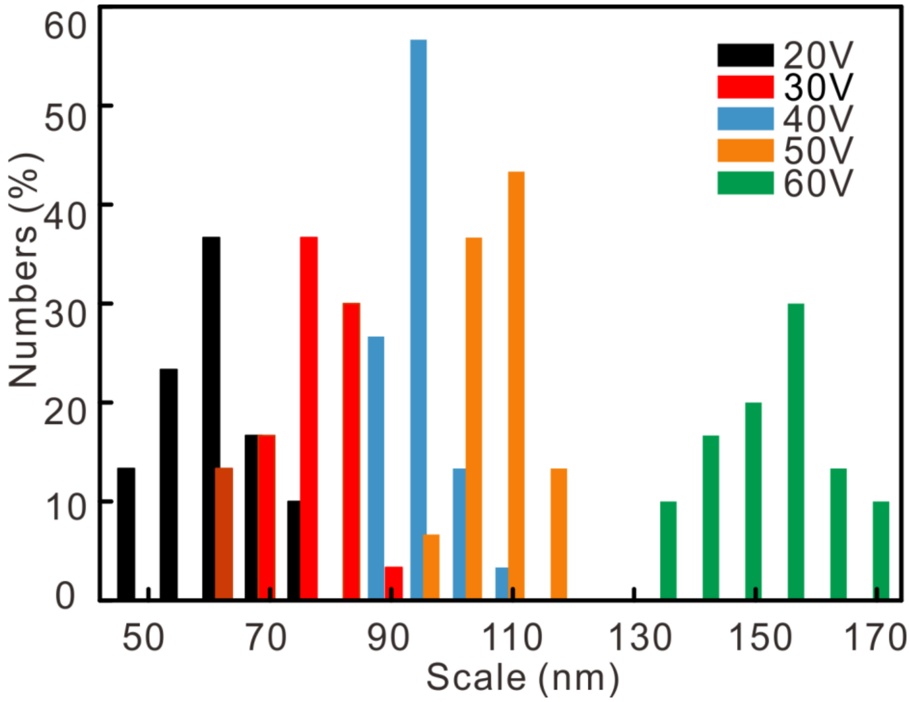

Supplementary Figure 8 | Statistics of the size of nanoparticles on rolled-up nanomembranes copied from different Al substrate after anodic oxidation with different voltages.

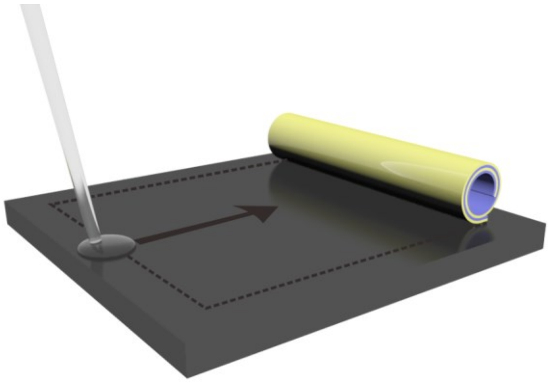

Supplementary Figure 9 | Schematic illustration of rolling architecture of delaminated prestrained layers manipulated by microdroplet.

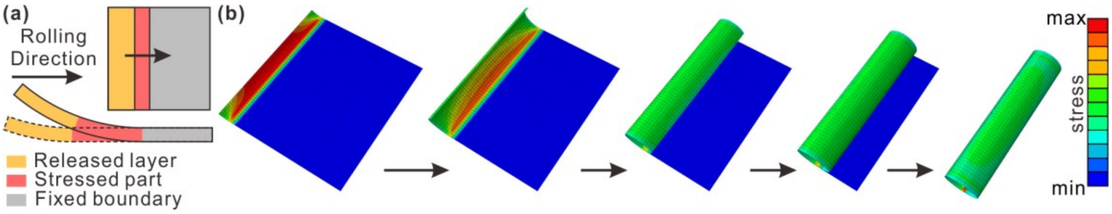

Supplementary Figure 10 | Model for quasi-static finite element analysis. (**a**) Simulation principles of quasi-static modeling (**b**) Simulated process of square-patterned nanomembrane rolling along its edge.

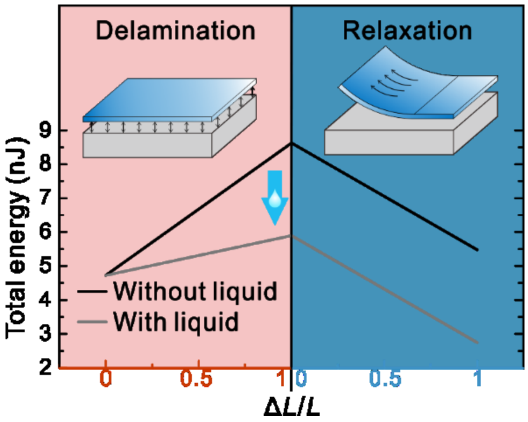

Supplementary Figure 11 | Calculation results of total energy related to relative released length (Δ*L*/*L*) in square nanomembrane. Black and red line is the energy variation before and after liquid treatment, respectively. Spontaneous delamination rolling process is divided into two separate parts as delamination and strain relaxation.

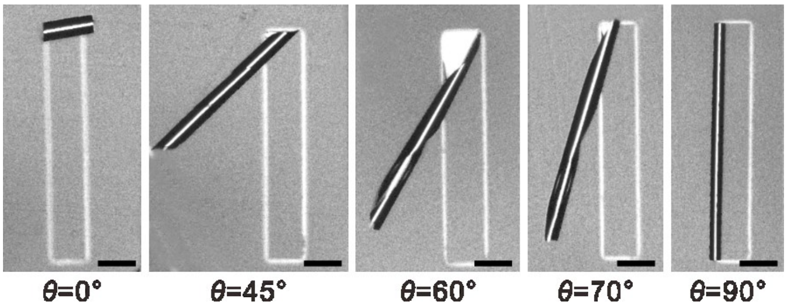

Supplementary Figure 12 | Precise control in rolling direction of rectangular-shaped nanomembrane. Scale bar, 50 µm.

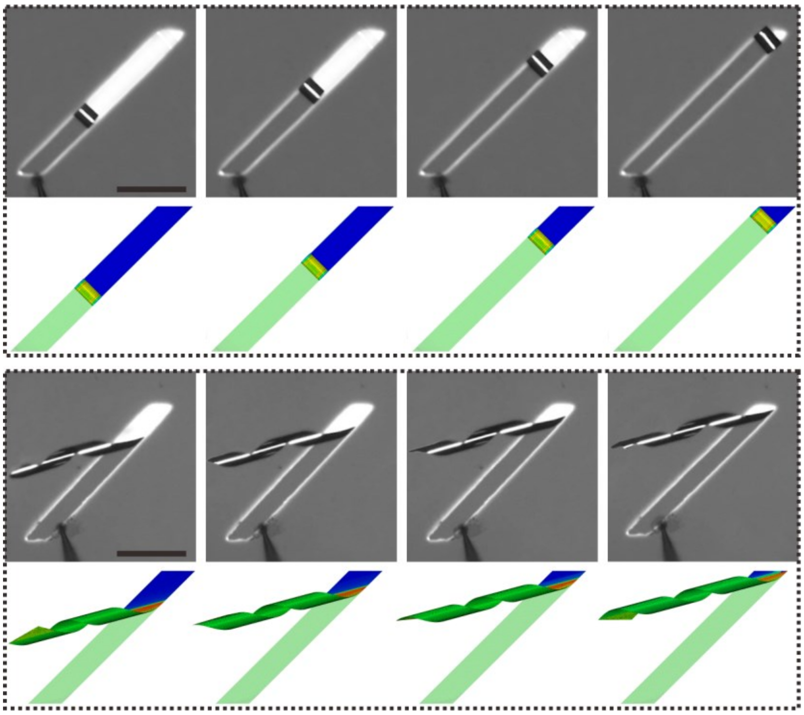

Supplementary Figure 13 | Simulated rolling process of microtube and microhelix, and corresponding experimental sequence. Scale bar, 100 µm.

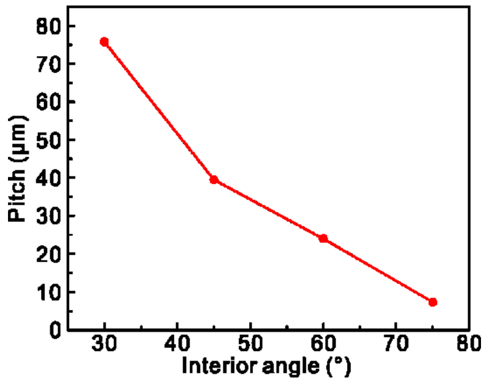

Supplementary Figure 14 | Helical pitch related to the interior angle of parallelogram pattern from Fig. 4a.

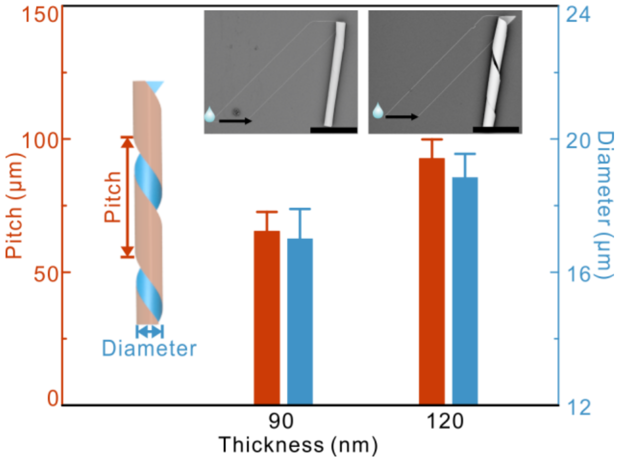

Supplementary Figure 15 | Pitch and diameter of rolled-up helical microstructures related to the total thickness. The scheme illustrates the definition of pitch and diameter. SEM images are corresponding helical microstructures where the droplet is the trigger point. Scale bar, 100 μm. Error bars represent SD.

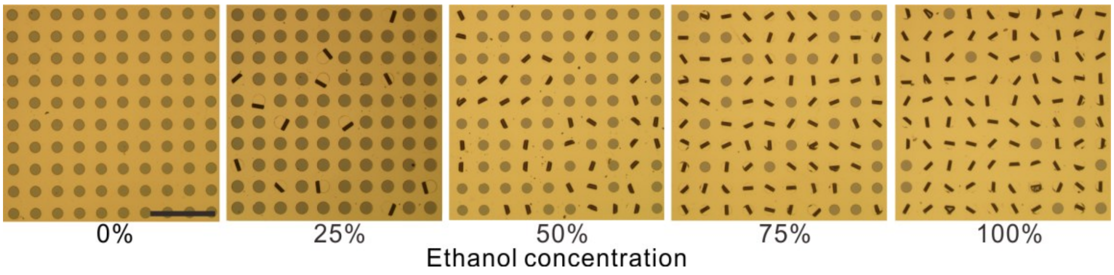

Supplementary Figure 16 | Optical images depicting the array of circle-patterned nanomembranes after placed in ethanol-water atmosphere with different concentration for 1 min. Scale bar, 500 μm.

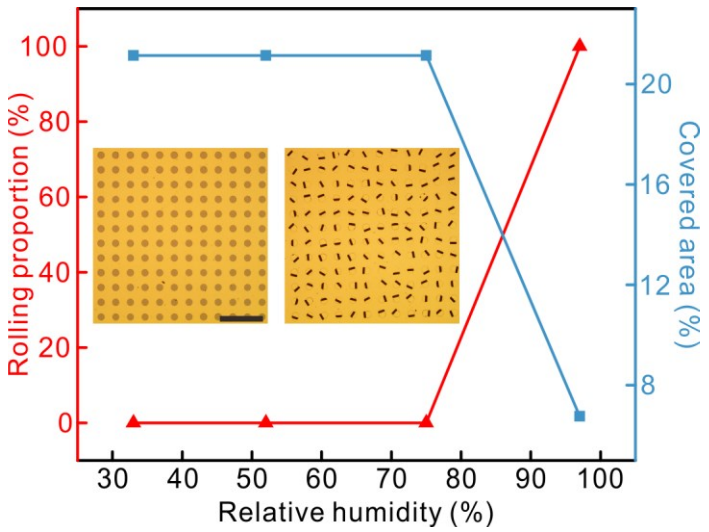

Supplementary Figure 17 | Humidity sensing by spontaneous rolling of patterned nanomembranes. Patterned nanomembranes consisting of SiO and Cr were deposited on Au substrate. The sample was placed in humid environment for 30 min and the humidity was obtained by different salt solutions. The insets present unrolled nanomembranes in low humidity environment and rolled-up nanomembranes in high humidity environment. Scale bar, 500 μm.

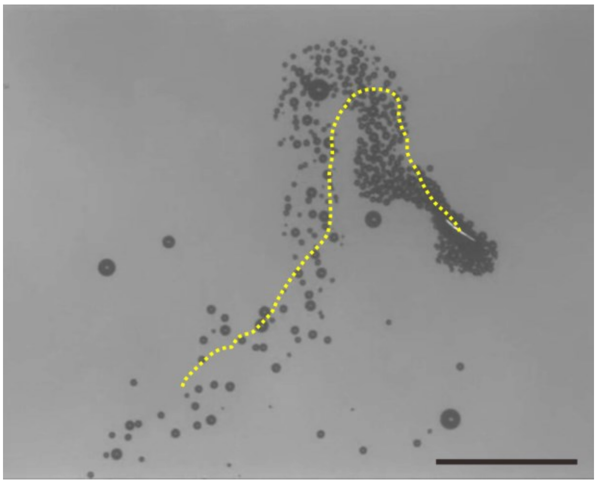

Supplementary Figure 18 | Optical image of single tubular micromotor in 5% hydrogen peroxide solution. Dashed line depicts the trajectory of the motion. Scale bar, 100 μm.

Supplementary Note 1 | Convenient methods for massive production from unpatterned nanomembranes

Rolling of unpatterned nanomembrane is established on the random cracks which create edges as the start position for rolling. A convenient method based on the liquid-triggered delamination is developed for massive fabrication of rolling microstructures, which is illustrated in Supplementary Figure 3a. First, selected pre-layer was deposited on the substrate to create van der Waals interaction without shadow mask. After deposition, one drop of liquid was dripped on the surface directly. The liquid intercalates into the defects of deposited nanomembrane, turning these defects into cracks. And further intercalation leads to the delamination of deposited nanomembrane, resulting in a large number of self-rolled-up microtubes. One instance was present in Supplementary Figure 3b in which Au/SiO/Fe rolling microstructures were fabricated on Al substrate. A number of microtubes were obtained among which we got some ultralong microtubes.

Moreover, an optional method with organic layer is illustrated in Supplementary Figure 4. The van der Waals interaction was created between the inorganic nanomembrane and polymer substrate (N,N-dioctyl-3,4,9,10-perylene tetracarboxylic diimide, PTCDI-C8). The polymer layer was deposited via thermal evaporation at 200 °C while the temperature of substrate was tuned as 150 °C. As the sample was cooled to the room temperature, shrinking of polymer layer due to thermal expansion generates nanocracks on the surface randomly. Continuous deposition of inorganic materials created van der Waals interaction with polymer. During the deposition process, this interaction is overcome by the strain gradient inside nanomembranes, leading to the spontaneous rolling starting from the nanocracks as illustrated in Supplementary Figure 5a. The SEM image in Supplementary Figure 5b presents randomly self-rolled-up Cr microtubes. Besides, nanocracks on

polymer layer were observed indistinctly, which is highlighted by red arrow.

Supplementary Note 2 | quasi-static FEM

Considering that the rolling of nanomembrane is guided by microdroplet, which is a history-dependent process, a transient quasi-static FEM modeling was applied to simulate the dynamic delamination and rolling behavior. The rationale of modeling is explained in Supplementary Figure 10a. The fixed boundary condition of multilayers (gray part) is moved along the guided direction step by step to release the nanomembrane. In every releasing step, a static modeling was used to simulate the large deformation generated by internal strain gradient (stressed part highlighted in red). Next step starts as previous simulation is finished. Supplementary Figure 10b presents simulated results toward the rolling process of a square-patterned bilayer, which was cut into 8 segments along its edge, to give a clear illustration of transient quasi-static FEM. With detailed partition of 2D pattern along the guided direction, the geometries of 3D microstructures could be modeled with excellent accuracy. Moreover, a quasi-dynamic rolling process can be modeled as we relate every step to time. Supplementary Figure 13 presents a set of intermediate modeling sequences and experimental rolling processes. Both tubular and helical microstructures were constructed from the rolling of parallelogram-patterned nanomembrane, and the simulated results match the whole rolling process well.

Supplementary Note 3 | Analytical calculation of elastic energy change during release of different patterned nanomembranes.

The calculation to analyze the energy variation during release is based on the plane stress assumption. As the parameters of layer i is set are thickness t_i, Young's modulus E_i, Poisson's ratio v_i and initial strain ε_i^0, the elastic energy density in a plane stress system can be written as

The surface density of energy can be calculated by integrating the elastic energy from the bottom to the top of nanomembrane

$$\bar{u} = \sum_{i=1}^{3} \int_{z_{i-1}}^{z_i} \frac{E_i}{2(1-v_i^2)} \left[\left(\varepsilon_{xi}^2 + \varepsilon_{yi}^2 \right) + 2 v_i \varepsilon_{xi} \varepsilon_{yi} \right] dz \tag{1}$$

$$z_0 = 0,\ z_1 = t_1,\ z_2 = t_2 + t_1,\ z_3 = t_3 + t_2 + t_1 \tag{2}$$

As for the nanomembrane without deformation, the strain is

$$\varepsilon_{xi} = \varepsilon_{yi} = \varepsilon_i^0 \tag{3}$$

As for the nanomembrane rolling along *x* axis, considering that there's no strain relaxation in *y* direction, this issue can be regarded as plane strain problem. So we have

$$\varepsilon_{yi} = \varepsilon_i^0, \varepsilon_{xi} = \varepsilon_i^0 + \varepsilon_{const} + \frac{z - z_b}{R} \tag{4}$$

For the strain in *x* direction, ε_{const} is the balanced strain after relaxation, referring to the strain change of neutral plane. And $\frac{z-z_b}{R}$ is the strain induced by geometric deformation, in which *z* is the *z* coordinate, z_b is *z* coordinate of neutral plane and *R* is the radius of bent nanomembrane. The value of ε_{const} and z_b are given by following equations.

$$\varepsilon_{const} = -\frac{\sum_{i=1}^{3} E_i^{'} t_i \eta_i \varepsilon_i^0}{\sum_{i=1}^{3} E_i^{'} t_i} \tag{5}$$

$$z_b = \frac{\sum_{i=1}^{3} E_i^{'} t_i (z_i + z_{i-1})}{2 \sum_{i=1}^{3} E_i^{'} t_i} \tag{6}$$

For plane strain case,

$$E_i^{'} = \frac{E_i}{1 - v_i^2}, \eta_i = 1 + v_i \tag{7}$$

The radius *R* can be solved by minimizing the elastic energy with respect to *R*. Then, the strain in *x*

direction can be calculated. As the pre-strained nanomembrane is regarded as isotropic materials, surface density of energy among the surface is considered equal everywhere ($\bar{u}_0$). Similarly, surface density after release is also regarded as equal ($\bar{u}_b$). Hence, the variation in total energy is generated from the difference between fixed and released parts. We assumed an ideal releasing process that the release boundary is fixed as a straight line perpendicular to the release direction, which is reasonable in the rolling towards one direction. Thus, the total elastic energy with released length *l* as defined in Fig. 2b can be written as:

$$E_{total} = \bar{u}_b \times S_{released} + \bar{u}_0 \times S_{fixed} \tag{8}$$

Supplementary Note 4 | Analytical calculation of total energy change during delamination and relaxation of nanomembrane

In Supplementary Note 3, the elastic energy of nanomembrane is discussed in detail. However, it's not enough to explain the spontaneous delamination and rolling behavior. In this note, the total energy is taken into consideration to see the variation related to the nanomembrane release.

In order to establish the total energy change of material system, the liquid-triggered rolling behavior was divided into two processes as illustrated in the insets of Supplementary Figure 11. The first process is the release of nanomembrane from substrate along rolling direction. And the second one is the rolling of nanomembrane. Energy variation in these two processes is adhesion energy and strain relaxation, respectively.

Here, the nanomembrane system same as Fig.2a is considered as an example, in which Au/SiO/Fe trilayer was deposited on glass substrate. And the pattern was designed as square with length of 100 μm. Rolling direction is set as one along the side of square. The energy change without liquid treatment is shown as black line in Supplementary Figure 11. In the first process, energy required

for the delamination of nanomembrane is the adhesion energy. This value between Au and SiO_2 (glass) is about 3.9 nJ[1]. And in the second process, the change of elastic energy is calculated as 3.0 nJ, which is smaller. This result reflects that the energy provided by strain relaxation is smaller than the energy required for delamination, which means the rolling of nanomembrane is not allowed.

On the other side, the adhesion between nanomembrane and substrate decreases to 20-30% due to liquid treatment[2]. Thus, the adhesion energy in the first process is reduced to 1.2 nJ, as depicted by red line in Supplementary Figure 11. Without liquid, the energy has the property of possessing two stable equilibria, both of which are natural equilibrium due to the existing energy barrier. With liquid treatment, the energy of strain relaxation is much larger than adhesion energy. As a result, this energy barrier is easier to be overcome through internal strain or disturbance from liquid. Once the hill of barrier is overcome, the structure transforms from planar to relaxed state spontaneously due to the lower energy in later one. This process in energy storage and releasing is similar to a snap-through behavior.

Supplementary Note 5 | Analytical calculation for the diameter of rolled-up microtubes

The model to illustrate the relationship between diameter of rolled-up microtubes and total thickness of nanomembranes is based on the plane strain assumption. The parameters needed for the calculation include the thickness t_i, Young's modulus E_i, Poisson's ratio v_i and initial strain ε_i^0 of the layer i.

According to the Supplementray Note 3, the diameter of rolled-up microtubes can be calculated as

$$Diameter = \frac{4\sum_{i=1}^{3} E_i^{'} t_i \left[z_i^2 + z_{i-1}z_i + z_{i-1}^2 - 3z_b(z_i + z_{i-1} - z_b)\right]}{3\sum_{i=1}^{3} E_i^{'} t_i (z_i + z_{i-1} - 2z_b)(c - \eta_i \varepsilon_i^0)} \tag{9}$$

In our situation, the thickness for each layer are the same ($t_1 = t_2 = t_3$). As we set the total thickness as t, the indirect parameters c and z_b can be rewritten as

$$c = \frac{E_1^{'}\eta_1\varepsilon_1^0 + E_2^{'}\eta_2\varepsilon_2^0 + E_3^{'}\eta_3\varepsilon_3^0}{E_1^{'} + E_2^{'} + E_3^{'}} \tag{10}$$

$$z_b = \frac{E_1^{'} + 3E_2^{'} + 5E_3^{'}}{6(E_1^{'} + E_2^{'} + E_3^{'})}t = z_c t \tag{11}$$

Then, the diameter can be rewritten as following:

$$Diameter = \frac{E_1^{'}(1 - 3z_c + 3z_c^2) + E_2^{'}(7 - 9z_c + 3z_c^2) + E_3^{'}(19 - 15z_c + 3z_c^2)}{E_1^{'}(1 - 2z_c)(c - \eta_1\varepsilon_1^0) + E_2^{'}(3 - 2z_c)(c - \eta_2\varepsilon_2^0) + E_3^{'}(5 - 2z_c)(c - \eta_3\varepsilon_3^0)}\frac{t}{3} \tag{12}$$

Therefore, it is proved that the diameter is proportional to the total thickness as the thickness of each layer are the same.

Supplementary References

1. Kennedy, M. S., Moody, N. R., Adams, D. P., Clift, M. & Bahr, D. F. Environmental influence on interface interactions and adhesion of Au/SiO_2. *Mater. Sci. Eng. A* **493**, 299-304 (2008).
2. Lee, C. H. et. al. Peel-and-stick: Mechanism study for efficient fabrication of flexible/transparent thin-film electronics. *Sci. Rep.* **3**, 2917 (2013).
