# Supplementary material for: Microdroplet-guided intercalation and deterministic delamination towards intelligent rolling origami
Source: Nat Commun. 2019 Nov 4;10:5019. doi: 10.1038/s41467-019-13011-w (PMC6828951; doi:10.1038/s41467-019-13011-w)
Supplement: Supplementary file 2 — Description of Additional Supplementary Files [file 41467_2019_13011_MOESM2_ESM.pdf]

Description of Additional Supplementary Files

Supplementary Movie 1 Microdroplet-guided rolling of square-patterned nanomembrane.

Supplementary Movie 2 Microdroplet-guided rolling of semicircle-patterned nanomembrane.

Supplementary Movie 3 Microdroplet-guided rolling of sector-patterned nanomembrane.

Supplementary Movie 4 Precise control in rolling direction of parallelogram-patterned nanomembrane.
